# Determinants and pattern of care seeking for preterm newborns in a rural Bangladeshi cohort

**DOI:** 10.1186/1472-6963-14-417

**Published:** 2014-09-22

**Authors:** Rashed Shah, Luke C Mullany, Gary L Darmstadt, Radwanur Rahman Talukder, Syed Moshfiqur Rahman, Ishtiaq Mannan, Shams El Arifeen, Abdullah H Baqui, on behalf of the ProjAHNMo Study Group in Bangladesh

**Affiliations:** Department of International Health, International Center for Maternal and Newborn Health (ICMNH), Johns Hopkins Bloomberg School of Public Health, Baltimore, Maryland USA; Department of Health and Nutrition, Save the Children-USA, Washington, DC USA; Global Development Division, Bill and Melinda Gates Foundation, Seattle, WA USA; International Centre for Diarrheal Disease Research, Bangladesh (icddr,b), Mohakhali, Dhaka, Bangladesh; Ma-Moni Project, MCHIP/Save the Children, Bangladesh Country office, Dhaka, Bangladesh

## Abstract

**Background:**

Despite the increased burden of preterm birth and its complications, the dearth of care seeking data for preterm newborns remains a significant knowledge gap. Among preterm babies in rural Bangladesh, we examined: 1) determinants and patterns of care seeking, and 2) risk analysis for care-seeking from qualified and unqualified providers.

**Method:**

Trained community health workers collected data prospectively from 27,460 mother-liveborn baby pairs, including 6,090 preterm babies, between June 2007 and September 2009. Statistical analyses included binomial and multinomial logistic regressions.

**Results:**

Only one-fifth (19.7%) of preterm newborns were taken to seek either preventive or curative health care. Among care-seeker preterm newborns, preferred providers included homeopathic practitioners (50.0%), and less than a third (30.9%) sought care from qualified providers. Care-seeking from either unqualified or qualified providers was significantly lower for female preterm babies, compared to male babies [Relative Risk Ratio (RRR) for unqualified care: 0.68; 95% Confidence Interval (CI): 0.58, 0.80; RRR for qualified care: 0.52; 95% CI: 0.41, 0.66]. Among preterm babies, care-seeking was significantly higher among caregivers who recognized symptoms of illness [RR: 2.14; 95% CI: 1.93, 2.38] or signs of local infection (RR: 2.53; 95% CI: 2.23, 2.87), had a history of child death [RR: 1.21; 95% CI: 1.07, 1.37], any antenatal care (ANC) visit [RR: 1.41; 95% CI: 1.25, 1.59]. Birth preparedness (RRR: 1.24; 95% CI: 1.09, 1.68) and any ANC visit (RRR: 1.73; 95% CI: 1.50, 2.49) were also associated with increased likelihood of care seeking for preterm babies from qualified providers.

**Conclusion:**

To improve care seeking practices for preterm babies and referral of sick newborns to qualified providers/facilities, we recommend: 1) involving community-preferred health care providers in community-based health education and awareness raising programs; 2) integrating postnatal care seeking messages into antenatal counselling; and 3) further research on care seeking practices for preterm babies.

**Electronic supplementary material:**

The online version of this article (doi:10.1186/1472-6963-14-417) contains supplementary material, which is available to authorized users.

## Background

Preterm newborns are at substantially higher risk for morbidity and mortality than full-term infants [1]. The burden of preterm birth and its complications have been increasing [2] and represent a significant issue in combating neonatal health risks and reducing neonatal mortality [3, 4], yet there is a paucity of research on care seeking for preterm newborns [5]. A few studies have reported behavioural aspects related to care-seeking practices [6–10], but often lack quantitative information on health care utilization, especially for preterm newborns. A systematic review on care-seeking for neonatal illness in low and middle income countries [5] unveiled a wide pattern for neonatal care seeking across study populations. In Bangladesh, studies have demonstrated that the proportion of newborns for whom care was sought from qualified providers (defined as doctors, nurse and paramedics trained to clinically practice western medicine) can vary substantially but generally is low (e.g. from 17% to 34%) [11–13].

Given the variability of socio-demographic and cultural contexts, differentials in perception of vulnerability or risk for newborns, and prevailing customs, traditions and beliefs within communities, it is critically important to understand community-specific patterns and determinants of population-level neonatal care seeking practices, especially for preterm newborns. Such data could help identify gaps and inform program approaches to promote care seeking for preterm babies [5, 14].

**Figure 1:**
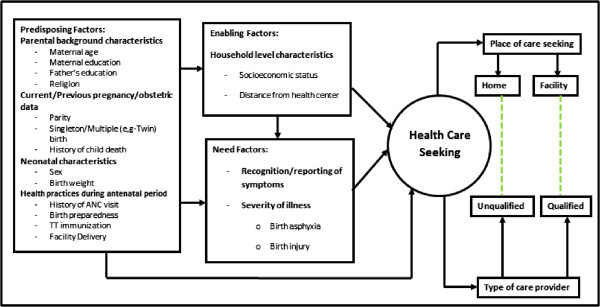
**Conceptual Framework.** *Adopted from*: [5, 18].

We aimed to examine the patterns and determinants of care seeking for preterm newborns and to conduct comparative risk analysis for care-seeking from qualified and unqualified providers of health care in a rural community in Bangladesh. As a complex interaction of multiple factors can cause delay in the decision to seek care [15–17], our approach is clarified through an adaptation of Andersen’s socio-behavioural model [18] of health services (Figure 1). We incorporated both 1) predisposing [maternal age, parental educational level, sex of the baby, previous obstetric history, birth order, antenatal care (ANC) status] and 2) enabling factors (socio-economic status, distance from a health facility) in the model, and assumed that an individual’s choice to seek health care is guided by these two types of factors. Other ‘need’ factors (e.g. recognition and perception of the need and severity) act as triggers on the decision which drive the individual to either seek care or refrain from seeking care [19] and are also included as independent variables in our analyses. Care-seeking for newborns, especially for preterm newborns, was additionally characterized by place of health care-seeking (home vs. facility). Finally, given the cultural norm of confinement or seclusion of both mother and baby until 40 days postpartum [20] in our area (and more broadly throughout South Asia), we also examined the care-seeking pattern from qualified vs. unqualified providers.

## Methods

### Study design

We analyzed prospectively collected data from a large community-based cluster-randomized trial (registered at ClinicalTrials.gov # NCT00434408) conducted in Sylhet district of Bangladesh to evaluate the impact of single or multiple (i.e. daily) cleansing of the umbilical cord with 4.0% chlorhexidine solution on overall neonatal mortality and incidence of cord infections. Details of the trial design have been published elsewhere [21].

### Study setting, population and implementation

The study was implemented in 22 unions (the smallest administrative unit with a health center) of Sylhet district in north-eastern Bangladesh during June 2007- September 2009. One female community health worker (CHW) was assigned for implementation of interventions and data collection from study participants in each of 133 geographical working units (“clusters”) within the study area.

Health care was available to the community within the study area through first-level health centers (each serving 20,000 population) and sub-district hospitals (each for ~200,000 population), neither of which were equipped to provide emergency care to newborns, especially preterm babies. Sylhet Medical College Hospital offered such specialized care but its location outside the study area requires approximately 2.5 hours to reach by bus.

### Study implementation

Bi-monthly pregnancy surveillance was conducted among all married women of reproductive age by house-to-house visits. Newly identified pregnant women were enrolled in the study, following agreement through an oral informed consent procedure. CHWs delivered a package of maternal and newborn health interventions (Additional file 1) and collected relevant data from all enrolled women at scheduled antenatal home visits (scheduled at ~12-16 and ~32 weeks gestational age). Enrolled women were followed through the end of pregnancy, and CHWs made scheduled visits at home during the postnatal period (days 0, 2, 5, 14, and 27) to assess the newborn using standardized tools.

### Assessment of independent variables

At enrollment, data were collected on age, literacy, religion, pregnancy history, and socio-demographic and economic information (educational attainment of women and husbands, household construction materials and assets). CHWs collected information on maternal care-seeking practices during antenatal home visits. They also assessed the family’s birth and newborn care preparedness (BNCP) status, reflected by practice of the following steps: selection of 1) a birth attendant and 2) newborn care personnel; arrangement for 3) clothes for newborn drying/wrapping, and 4) emergency transport, if needed; 5) allocating emergency savings; and 6) possession of a clean delivery kit (CDK). BNCP status was categorized as “fully compliant” (all 6 of the above-mentioned steps were reported as practiced), “partially compliant” (practiced 1–5 steps), or “non-compliant” (0 steps taken).

At the first postnatal home visit, CHWs collected basic data on labor and delivery, date/time of birth, and sex of the baby. At all postnatal visits, additional data on immediate essential newborn care practices (bathing, drying, wrapping, breastfeeding), reported morbidity, and vital status of the child were collected.

### Primary exposure variable

LMP date was recorded at the enrollment visit and maternal recall was facilitated by using calendars and memory aids. Some women could not remember/report their LMP date and some women became pregnant during the postpartum amenorrheic period and thus could not provide an LMP date. For those with available LMP estimate, gestational age at birth (in completed weeks) was computed by subtracting the reported date of the first day of the last menstrual period (LMP) from the date of birth.

### Assessment of outcome variable

The primary outcome in this study was “care seeking”. Care seeking was defined as any care (either preventive or curative) sought from any health care provider (either qualified or non-qualified) for a newborn. Relevant operational definitions and health care provider categories are listed in Additional file 2.

### Statistical analyses

Our analyses included all reported live births within the study area during the study period who received a CHW’s assessment visit during the first two weeks of life. We excluded women who, at the time of enrollment, could report neither an LMP date nor the duration (in month/day) since her last menstruation, as this estimate was required to define gestational age for each live-born baby.

The broad ‘care seeking’ variable was further categorized as: 1) sought no care, and those who sought care from 2) unqualified providers, or 3) qualified providers. We treated these categories following the above mentioned hierarchical order and the highest category was considered in case of seeking care from multiple categories of health care providers (for example if a baby sought care from unqualified providers on the first attempt and later sought care from a qualified provider, this baby was counted as a care seeker from a qualified provider).

Preterm was identified as birth before 37 completed weeks of gestation, or fewer than 259 days since the first day of the LMP [22]. Adapted from previous studies and existing literature [2, 23, 24], preterm births were sub-categorized as (1) Very preterm (28–31 weeks of gestation), (2) Moderate preterm (32–34 weeks of gestation) and (3) Late preterm (35–36 weeks of gestation). Births at ≥37 weeks were classified as term births. Following International Classification of Disease (10^th^ Revision) [25], all newborns with any sign of life at birth were recorded as live births.

Wealth index score [26] was constructed for each household by principal component analysis of basic housing construction materials (e.g. construction materials for the wall, roof, and floor) and household assets. We also estimated the straight line distance between nearest health facility and household by using location coordinates (longitude/latitude) for households and health facilities, collected by using global positioning system.

Percent distributions of term and preterm babies were computed by their care seeking status (from nonqualified and qualified providers or for non-care seekers). Crude associations between potential determinants for seeking care for preterm babies were modeled using binomial regression analysis with generalized linear model, by using log link (or a poisson model in case of convergence failure) [27–29]. To account for clustering, standard errors were adjusted using the generalized estimating equation approach with exchangeable correlation structure [30, 31]. Factors associated with choice of providers were examined using multinomial logistic regression which is widely used for modeling polychotomous outcomes including health seeking behaviors [32–34]. “Hotdeck” method by cluster [35] was used to impute missing data for ‘birth preparedness status’ and ‘any ANC visit’ variables. Analyses were conducted using STATA (version 12.1) [36].

### Ethical approval

We received ethical approval from the Johns Hopkins Bloomberg School of Public Health Institutional Review Board and the Ethical Review Committee of the International Centre for Diarrhoeal Disease Research, Bangladesh.

## Results

**Figure 2:**
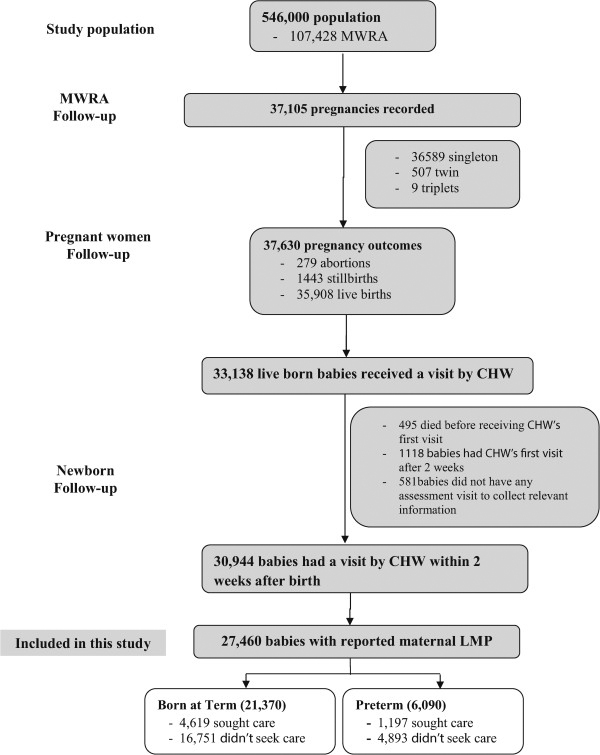
**Study profile.**

Between June 2007 and September 2009, we recorded 37,630 pregnancy outcomes and 35,908 live births within the study area. Of these, 27,460 mother-live born baby pairs (including 6,090 preterm babies) were analysed in this study (Figure 2). Most of the respondent women (89%) were able to report their LMP date, and were included in the analyses.

### Determinants of seeking care (either curative or preventive) for preterm newborns

#### Predisposing factors

Maternal age, parental education and religion were not associated with care seeking for preterm newborns. Among all the reported preterm births, 46.7% were female. Compared to male preterm babies, caregivers of female preterm newborns were 27% less likely to seek care (Relative Risk (RR): 0.73; 95% Confidence Interval (CI): 0.66, 0.80). There was no difference in care seeking for preterm babies born from multiple compared to singleton pregnancies (RR: 1.12; 95% CI: 0.92, 1.35).

Among women who delivered preterm births, more than a quarter (28.6%) had previously experienced the death of one of their children (any child born alive and died later). Table 1 shows a small, but statistically significant association was observed between likelihood of care-seeking for a preterm infant and history of a previous child death (RR: 0.96; 95% CI: 0.93, 0.98).

**Table 1:** **Determinants of seeking care (either curative or preventive) for preterm newborns**

Variables	Newborns included in the study N = 27,460		Preterm newborns who sought care		Unadjusted risk ratio (95% CI)	Adjusted ^€^ risk ratio (95% CI)
	Term newborns	Preterm newborns				
	N = 21,370	N = 6,090	N = 1,197			
	(%)	(%)	n	% (row)		
**Predisposing factors**						
**Mother’s age**						
<25 years	33.5	30.1	418	22.8	Ref	Ref
25-29 years	33.7	33.5	371	18.2	0.80 (0.69, 0.92)*	0.95 (0.82, 1.09)
30-34 years	20.3	22.0	251	18.7	0.82 (0.70, 0.96)*	0.96 (0.80, 1.14)
35 years & above	12.5	14.5	157	17.8	0.78 (0.65, 0.94)*	0.92 (0.75, 1.13)
**Mother’s education**						
Below primary	47.5	57.3	609	17.5	Ref	Ref
Primary and above	52.5	42.7	588	22.6	1.29 (1.15, 1.45)*	1.08 (0.96, 1.22)
**Father’s education**						
Below primary	55.6	64.3	710	18.1	Ref	Ref
Primary and above	44.4	35.7	487	22.4	1.24 (1.10, 1.39)*	1.02 (0.91, 1.15)
**Religion**						
Islam	95.4	95.7	1151	19.8	Ref	Ref
Others	4.6	4.3	46	17.6	0.89 (0.66, 1.19)	1.03 (0.79, 1.34)
**Single/Multiple birth**						
Singleton	98.3	95.4	1108	19.1	Ref	Ref
Multiple birth	1.7	4.6	89	31.8	1.67 (1.34, 2.07) *	1.12 (0.92, 1.35)
**History of child death**						
No	75.7	71.4	833	19.2	Ref	Ref
Yes	24.3	28.6	364	20.9	1.09 (0.96, 1.23)	1.21 (1.07, 1.37)*
**Sex of the baby**						
Male	51.3	53.3	737	22.7	Ref	Ref
Female	48.7	46.7	460	16.2	0.71 (0.63, 0.80)*	0.73 (0.66, 0.80)*
**Birth preparedness status**						
Not compliant	3.3	21.8	276	20.8	Ref	Ref
Partially compliant	62.7	51.2	544	17.4	0.84 (0.72, 0.97)*	0.91 (0.81, 1.03)
Fully compliant	34.0	27.0	377	22.9	1.10 (0.94, 1.29)	1.14 (0.99, 1.30)
**Any ANC visit**						
No	40.3	49.8	409	13.5	Ref	Ref
Yes	59.7	50.2	788	25.8	1.92 (1.70, 2.16)*	1.41 (1.25, 1.59)*
**Enabling factors**						
**Wealth quintile**						
Lowest (Poorest)	18.6	23.4	195	13.7	Ref	Ref
Second lowest	19.0	23.5	247	17.3	1.26 (1.05, 1.52)*	1.18 (1.00, 1.39)
Middle quintile	19.6	21.4	282	21.6	1.58 (1.31, 1.89)*	1.38 (1.17, 1.64)*
Second highest	20.4	18.5	246	21.9	1.60 (1.33, 1.93)*	1.34 (1.13, 1.60)*
Highest (Richest)	22.3	13.2	227	28.3	2.07 (1.71, 2.51)*	1.57 (1.29, 1.90)*
**Distance from health facility**						
Less than 2 Km	49.6	48.9	695	23.3	Ref	Ref
2-2.99 Km	29.6	30.0	304	16.6	0.71 (0.62, 0.82)*	0.79 (0.70, 0.88)*
3 Km or more	20.8	21.1	198	15.4	0.66 (0.57, 0.77) *	0.75 (0.66, 0.87) *
**Need factors**						
**Birth Asphyxia**						
No	88.5	89.3	989	18.2	Ref	Ref
Yes	11.5	10.7	208	32.0	1.76 (1.51, 2.04)*	1.28 (1.12, 1.45)*
**Any signs of injury at birth**						
No	98.0	98.2	1152	19.3	Ref	Ref
Yes	2.0	1.8	45	40.9	2.12 (1.58, 2.86)*	1.44 (1.13, 1.84)*
**Symptoms of illness**						
No	84.1	82.5	767	15.3	Ref	Ref
Yes	15.9	17.5	430	40.3	2.64 (2.34, 2.97)*	2.14 (1.93, 2.38)*
**Signs of local infections**						
No	94.9	95.5	1031	17.7	Ref	Ref
Yes	5.1	4.5	166	60.6	3.42 (2.90, 4.03)*	2.53 (2.23, 2.87)*

*p < 0.05;

^€^Adjusted for all other covariates including maternal age, education of women and their husbands, religion, wealth score, distance from nearby health facility, number of babies delivered, birth weight and sex of the newborn, signs/symptom of illness/infection, birth asphyxia, birth injury, history of child death, birth preparedness, ANC visit, TT immunization.

Caregivers who had any ANC visit were 41% more likely to seek care for their preterm baby (RR: 1.41; 95% CI: 1.25, 1.59). Likelihood of care-seeking was not significantly different among caregivers who had “Fully compliant” BNCP status (RR: 1.14; 95% CI: 0.99, 1.30) compared to those who were “non-compliant”.

#### Enabling factors

In Table 1, we found that higher socioeconomic status was associated with increased likelihood of care-seeking for preterm babies. Respondents in the richest group were >1.5 times more likely to seek care compared to the respondents in the poorest group [RR: 1.57; 95% CI: 1.29, 1.90]. There was lower likelihood of care seeking for preterm babies from households further from facilities; compared to babies from households within 2 km of a health facility, preterm babies born >2 km from a health facility were 25% less likely to seek care (RR: 0.75; 95% CI: 0.66, 0.87).

#### Need factors

Among preterm babies, 17.5% had reported symptoms of illness and 10.7% suffered birth asphyxia; signs of local infection were found among 4.5%, and only a few (1.8%) had signs of birth injury (Table 1). Care-seeking for preterm babies was >2-fold significantly higher among caregivers who had recognized symptoms of illness (RR: 2.14; 95% CI: 1.93, 2.38), or signs of local infections (RR: 2.53; 95% CI: 2.23, 2.87). Preterm infants who suffered birth asphyxia (RR: 1.28; 95% CI: 1.12, 1.45) or who had any birth injury (RR: 1.44; 95% CI: 1.13, 1.84) were similarly found to have higher likelihood to be taken for care-seeking.

#### Pattern of care seeking

Analysing overall care seeking practice among all babies (term and preterm) revealed (in Table 2) that parents/families of 21,644 (78.8%) newborns sought ‘no care’, and care-seeking was significantly lower (p < 0.01) among preterm newborns (1,197/6,090; 19.7%) compared to babies born at term (4,619/21,370; 21.6%). Among all care-seekers, less than a third (32.8%) of newborns sought care from qualified providers. The preferred health provider for neonatal care seeking was homeopathic practitioners (49.6%) followed by qualified medical doctor (21.8%). Provider preference was similar for term and for preterm infants, irrespective of preterm birth categories.

**Table 2:** **Distribution of newborns seeking curative and preventive care from different types of providers, by gestational age**

Type of providers	Health care Providers	Newborns for whom families/parents sought care health care ^¥^			
		Babies born at Term (>37 weeks) and sought care	Babies born preterm and sought care (N = 1,197)		
			Very preterm (28–31 weeks)	Moderate preterm (32–34 weeks)	Late preterm (35–36 weeks)
		N = 4,619	N = 135	N = 411	N = 651
Qualified	Doctor [Medical graduate (MBBS^1^)]	1025 (22.2)	26 (19.3)	98 (23.8)	118 (18.1)
	Nurse/Paramedic (FWV/MA/SACMO^1^)	514 (11.1)	18 (13.3)	43 (10.5)	67 (10.3)
Non-Qualified	HA/FWA^1^	66 (1.4)	2 (1.5)	6 (1.5)	17 (2.6)
	CHW^1^	99 (2.1)	6 (4.4)	10 (2.4)	22 (3.4)
	Homeopath	2288 (49.5)	62 (45.9)	187 (45.5)	350 (53.8)
	Village Doctor	387 (8.4)	12 (8.9)	40 (9.7)	46 (7.1)
	Others*	240 (5.2)	9 (6.7)	27 (6.6)	31 (4.8)

^¥^Figures are presented as numbers (percent); *χ* ^2^ = 6.66; p = 0.084.

^*^Ayurvedic, quack (Ojha, Kabiraj), Traditional Birth Attendant (TBA), Trained Traditional Birth Attendant (TTBA), herbal practitioner, Spiritual Leader/Imam (Muslim religious leader).

^1^ *MBBS* = Bachelor of Medicine and Bachelor of Surgery; *FWV* = Family Welfare Visitor; *SACMO* = Sub-Assistant Community Medical Officer; *MA* = Medical Assistant; *HA* = Health Assistant; *FWA* = Family Welfare Assistant; *CHW* = Community Health Worker.

#### Results from multinomial logistic regression analysis

Estimated relative rate ratios (RRRs) for care seeking compared to not-seeking care among preterm newborns are presented in Table 3. Only father’s educational status and sex of the child were significantly associated with care seeking from qualified (but not unqualified) providers as opposed to not seeking any care. Babies of fathers with five or more years of schooling compared to less than five years of schooling or no schooling were 1.33 times (RRR: 1.33; 95% CI:1.02, 1.74) more likely to seek care from qualified providers. The RRR of seeking care from both qualified and unqualified providers was significantly lower for female babies compared to male babies (RRR for qualified care: 0.52; 95% CI: 0.41, 0.66; RRR for unqualified care: 0.68; 95% CI: 0.58, 0.80). Previous history of child death was associated with higher use of both of qualified and non-qualified care (RRR of qualified care: 1.52; 95% CI: 1.12 – 2.05; RRR of non-qualified care: 1.24; 95% CI: 1.02 – 1.50). Complete birth preparedness (RRR: 1.24; 95% CI: 1.09 - 1.68) and any ANC visit (RRR: 1.73; 95% CI: 1.30 – 2.30) increased the likelihood of care seeking from a qualified provider for preterm babies.

**Table 3:** **Multinomial logistic regression** ^**$**^ **analysis for care seeking (preventive or curative) for preterm newborns**

	Non-qualified care provider ^**^		Qualified provider ^*^	
	Relative Risk Ratio (RRR)	95% CI	Relative Risk Ratio (RRR)	95% CI
**Parental characteristics**				
**Mother’s age**				
<25 years	Ref		Ref	
25-29 years	0.86	0.69 – 1.08	0.93	0.66 – 1.31
30-34 years	0.76	0.56 – 1.01	1.51	1.01 – 2.27^∞^
35 years & above	0.78	0.57 – 1.08	1.11	0.67 – 1.82
**Mother’s education**				
Below primary	Ref		Ref	
Primary and above	1.14	0.94 – 1.39	1.04	0.78 – 1.38
**Father’s education**				
Below primary level	Ref		Ref	
Primary and above	0.92	0.76 – 1.11	1.33	1.02 – 1.74^∞^
**Religion**				
Islam	Ref		Ref	
Others	1.00	0.66 – 1.51	1.00	0.55 – 1.84
**Household characteristics**				
**Household wealth quintile**				
Lowest quintile (Poorest)	Ref		Ref	
Second lowest quintile	1.22	0.95 – 1.56	1.33	0.89 – 1.98
Middle quintile	1.63	1.25 – 2.11^∞^	1.53	1.02 – 2.30^∞^
Second highest quintile	1.44	1.09 – 1.90^∞^	1.75	1.15 – 2.64^∞^
Highest quintile (Richest)	1.58	1.15 – 2.19^∞^	2.84	1.82 – 4.42^∞^
**Distance from health facility**				
Less than 2 Km	Ref		Ref	
2-2.99 Km	0.78	0.65 – 0.94^∞^	0.53	0.40 – 0.70^∞^
3 Km or more	0.82	0.67 – 1.01	0.36	0.25 – 0.52^∞^
**Index pregnancy and previous obstetric related characteristics of the mother**				
**Single/multiple birth**				
Singleton	Ref		Ref	
Multiple birth	0.98	0.66 – 1.45	1.47	0.95 – 2.28
**History of child death**				
No	Ref		Ref	
Yes	1.24	1.02 – 1.50^∞^	1.52	1.12 – 2.05^∞^
**Characteristics of the newborn**				
**Sex of the baby**				
Male	Ref		Ref	
Female	0.68	0.58 – 0.80^∞^	0.52	0.41 – 0.66^∞^
**Birth Asphyxia**				
No	Ref		Ref	
Yes	1.45	1.15 – 1.84^∞^	1.69	1.23 – 2.31^∞^
**Any signs of injury at birth**				
No	Ref		Ref	
Yes	1.39	0.82 – 2.38	2.97	1.62 – 5.45^∞^
**Reported symptoms of illness**				
No	Ref		Ref	
Yes	3.18	2.64 – 3.83^∞^	3.50	2.71 – 4.53^∞^
**Signs of local infections**				
No	Ref		Ref	
Yes	6.53	4.82 – 8.84^∞^	5.06	3.35 – 7.65^∞^
**Health practices during antenatal period**				
**Birth Preparedness**				
Not compliant	Ref		Ref	
Partially compliant	0.90	0.73 – 1.10	0.83	0.61 – 1.11
Fully compliant	1.17	1.04 – 1.47^∞^	1.24	1.09 – 1.68^∞^
**Any ANC visit**				
No	Ref		Ref	
Yes	1.54	1.28 – 1.86^∞^	1.73	1.30 – 2.30^∞^

^$^Reference category “No care received”. ^∞^p <0.05.

*Doctor (medical graduate), nurse, paramedic (Family Welfare Visitor, sub-assistant community medical officer) are considered as qualified provider.

**All other providers.

Household wealth quintile and distance from nearest health facility were significantly associated with care seeking, especially from qualified but also from non-qualified providers. All the need factors (except birth injury) in the model were significantly associated with using qualified and nonqualified care.

## Discussion

Our findings confirm that parents and caregivers in rural Bangladesh are reluctant to seek care for preterm babies; among those who seek care, they prefer to consult with unqualified rather than qualified providers. Preterm neonates are especially vulnerable to temperature instability, feeding difficulties, low blood sugar, infections and breathing difficulties - conditions which pose a critical need for care seeking for preterm babies. The low rate of care seeking for preterm babies (19.6%) in our study is consistent with findings from previous research in Bangladesh, Nepal, Pakistan and India [15].

Homeopathic practitioners are the preferred care providers for preterm infants in our study population. An earlier study conducted in same study area [20] reported similar parental preference for homeopathic care for their newborns over qualified and other non-qualified care providers. Homeopaths are mostly self-educated, but some possess recognized qualifications from government and/or private homeopathic colleges [37]. Both health care providers and parents often agree that very small babies or babies born too soon, irrespective of disease status, are ‘high risk’ (biomedical term) or ‘vulnerable’ (approximate translation of local terms). The difference lies in what is seen as appropriate treatment for vulnerable infants. Parents may perceive that biomedical treatments such as injections and antibiotics are too strong, and that vulnerable infants cannot withstand them. Parental preference for homeopathic providers is possibly because homeopathic medicine is thought to exert slow and gentle effects, which is perceived to be more acceptable than ‘strong’ modern medicines for a vulnerable baby.

Paternal education and sex of the newborn were significantly associated with care seeking from a qualified provider while mother’s education was not. Although mothers take most health related decisions at home regarding diarrheal disease and immunizations [38], fathers are more likely to take decisions for seeking care outside the home in rural Bangladeshi society [39]. Sex differential in care seeking has been reported in previous studies in South Asia [12, 40]. Consistent with strong son preference in this region [41, 42] and as reported in a study in rural India [43, 44], our study also revealed that female babies are less likely to be taken for qualified medical care compared to male babies.

We found that household wealth status and distance from the nearest health facility were significantly associated with care seeking from qualified providers in our study population, which is also consistent with previous reports [20]. Household economic status is an established factor associated with care seeking for children [12, 40, 45–50]. Distance from health facilities has also been an important barrier to health care access, including child health services, in other settings [50–52].

We found significant associations between positive health behaviours and antenatal practices (e.g., ANC visit, BNCP) and care seeking for preterm babies from a qualified or unqualified provider. Moreover, similar to previously reported results [45, 53], we also found a significant increase in the probability for care seeking for preterm newborns when signs of illnesses (asphyxia, birth injury, local infections including skin and eye infections and oral thrush) are recognized by parents or caregivers. These signs are visible, which may make family members more worried. Current evidence also shows that recognition of early danger signs of neonatal infections improves timely care seeking [54]. The strategy of study intervention delivery which included recognition of signs of illnesses among newborns by conducting assessments during postnatal home visits by trained CHWs, making referral and providing support to families for referral compliance also might explain such increased probability of care seeking for preterm newborns with signs of injury and infections. Our results reiterate an urgent need to educate parents/caregivers on recognition of risk factors and danger signs for mortality and morbidity for preterm babies. Although improved recognition of illness signs has been associated with increased care seeking in sick children [8, 55–58], other socio-cultural factors are interlinked with decisions to seek care from a qualified provider [6, 20, 59, 60]. For example, taking a sick infant outside the home is often perceived by the parents/caregivers as exposing the baby to increased risk of encountering malevolent spirits or the glare (“evil eye”) of jealous neighbours, which is believed to be the source of illness [12, 20]. In Sylhet, the concept of malevolent spirits is prevalent (locally called *‘upri’*) and is believed to manifest in neonatal illnesses characterized by high fever, crying, not wanting to eat, black spots on the skin, unusual quietness and strange facial expressions. Similar effects are perceived as the outcome of a glare from a jealous neighbour (locally named as *‘nazar’*) [20].

We enrolled a large number of mother-live born baby pairs and followed all live births through the neonatal period. Prospective design of this study eliminated the risk of selection as well as recall biases which are common in cross sectional and retrospective studies. Most of the known factors associated with care seeking practice were adjusted for in the analysis. However, a major limitation of the study was our reliance on LMP to determine gestational age. Common criticisms of the LMP method for gestational age determination include possible inaccuracy in recall, heaping on certain dates, and generalized assumption of “normal” menstrual cycle [61–63]. Given the need for clinical skills to determine gestational age by Dubowitz or Ballard methods and technical skills plus costs in using ultrasound, LMP remains the most feasible option in many rural, low resource settings such as ours. A related concern is the potential threat of having selection bias due to exclusion of women who could not report their LMP date. We examined for any differential in the characteristics of women whom we excluded, and found nothing significant. By restricting analyses to newborns visited by a CHW within two weeks after the birth, we may have potentially introduced survival bias; for example by excluding a baby who died before receiving a CHW visit (n = 495; 1.8% of 27,460).

## Conclusions

Our study results yielded the following recommendations to improve health care seeking for preterm babies in similar settings: 1) Involve community-preferred health care providers, even if they are unqualified (i.e.- not qualified in terms of western medicine practice; for example: homeopathic practitioners), to facilitate community-based health education and awareness raising programs; consider training them to recognize signs of illness and to refer sick newborns to qualified providers/facilities; and 2) Integrate postnatal care seeking messages (for both mother and baby) into antenatal counselling. Simultaneously, community-based health counselling and behaviour change communication strategies might have the potential to improve parental recognition of illness leading to early health care seeking for newborns, specifically preterm babies, and thus possibly will be critical for achieving success in community-based maternal and newborn health programs in low-income countries. Finally, we recommend further studies on community-level care seeking practices for preterm babies which would help in planning programs to reduce morbidity and mortality risks for babies who are ‘Born Too Soon’.

## Authors’ information

ProjAHNMo stands for Project for Advancing Health of Newborn and Mothers.

## Electronic supplementary material

Additional file 1: **Basic Prenatal Maternal and Newborn Care Package [web-only].** (DOCX 15 KB)

Additional file 2: Operational Definitions** **[web-only].** (DOCX 19 KB)
