# Supplementary material for: Determinants and pattern of care seeking for preterm newborns in a rural Bangladeshi cohort
Source: BMC Health Serv Res. 2014 Sep 22;14:417. doi: 10.1186/1472-6963-14-417 (PMC4261985; doi:10.1186/1472-6963-14-417)
Supplement: Supplementary file 1 — Additional file 1: Basic Prenatal Maternal and Newborn Care Package [web-only]. (DOCX 15 KB) [file 12913_2014_3643_MOESM1_ESM.docx]

**Additional file 1: Basic Prenatal Maternal and Newborn Care Package [web-only]**

| Counselling/messages and demonstration |
| --- |
| - Antenatal care - Tetanus toxoid immunization - Recognition of maternal danger signs during pregnancy, delivery and postnatal - Pre-selection of birth attendant - Clean and hygienic delivery - Immediate and exclusive breastfeeding - Thermal care of the newborn - Recognition of postnatal neonatal danger signs - Care seeking for maternal and newborn danger signs - Demonstration on use of birthing kit |
| Supplements and supplies |
| - Iron and folic acid supplements - Clean birthing kit |
