# Supplementary material for: Determinants and pattern of care seeking for preterm newborns in a rural Bangladeshi cohort
Source: BMC Health Serv Res. 2014 Sep 22;14:417. doi: 10.1186/1472-6963-14-417 (PMC4261985; doi:10.1186/1472-6963-14-417)
Supplement: Supplementary file 2 — Additional file 2: Operational Definitions** [web-only]. (DOCX 19 KB) [file 12913_2014_3643_MOESM2_ESM.docx]

**Additional file 2: Operational Definitions^**^ [web-only]**

- A caregiver was defined as the individual who looked after the newborn and was often a family member, such as the mother or grandmother.
- We defined ‘Qualified’ health care provider as a medically trained provider, one who practiced western medicine, was employed at a governmental health facility, or as a nongovernmental consultant at a privately owned chamber, clinic and/or hospital. Medical graduate doctors and specialists, nurses, and paramedics [Family Welfare Visitor (FWV), Medical Assistant (MA) and Sub-Assistant Community Medical Officer (SACMO)] were included in our analyses as ‘Qualified’ health care providers. Qualified providers within public sector offer care from a government facility, and receive a fixed salary and thus have less incentive to focus on care recipients.
- All other providers (e,g., birth attendant with or without training, homeopathic practitioners, village doctors, ayurvedic practitioners, traditional healers, spiritual healers, and salespeople/dispensers at drug store/pharmacy) who are mostly self-educated and self-employed health care providers who use to dispense medicines without prescriptions are defined as ‘Unqualified’ providers. We also considered frontline health workers, including Health Assistant, Family Welfare Assistant and community health workers as non-qualified providers. These health workers usually receive basic training on community health related topics and are employed in public and private sector to conduct community based surveillance, home-based health education and counselling and door-step delivery of targeted health services including referral.
- Homeopaths are mostly self-educated; some possess recognized qualifications from government or private homeopath colleges. They practice alternative medicine following Samuel Hahnemann’s pharmacopeia.
- Village doctors (also known as rural medical practitioners) mostly received (few weeks- few months) on common illnesses from semi-formal private institutions those are unregistered and unregulated and do not follow any standard curriculum. Very small proportion of these village doctors received 12 months training from a short-lived government sponsored program in 1980s (*Palli Chikitsok* training program after *Barefooted doctors* model in Mao’s China)
- Most of the drugstore salespeople/ drug dispenser have had no training in drug dispensing, not to mention training in diagnosis and treatment
- Traditional healers’ (called kabiraj) practice is mostly based on diet, herbs and exercise
- Spiritual healers (called Ojha) use sacrificed water, oil or chant religious verses for healing. Religious leaders (e, g- Imam, Purohit) also similarly serve as non-secular faith healers.

*^**^ Adapted from previous studies [1-3]*
